# Interleukin 11 (IL-11): Role(s) in Breast Cancer Bone Metastases

**DOI:** 10.3390/biomedicines9060659

**Published:** 2021-06-08

**Authors:** Paola Maroni, Paola Bendinelli, Anita Ferraretto, Giovanni Lombardi

**Affiliations:** 1 Laboratory of Experimental Biochemistry & Molecular Biology, IRCCS Istituto Ortopedico Galeazzi, Via R. Galeazzi 4, 20161 Milano, Italy; anita.ferraretto@unimi.it (A.F.); or lombardi@awf.poznan.pl (G.L.); 2 Dipartimento di Scienze Biomediche per la Salute, Università degli Studi di Milano, Via L. Mangiagalli 31, 20133 Milano, Italy; paola.bendinelli@unimi.it; 3 Department of Athletics, Strength and Conditioning, Poznań University of Physical Education, Królowej Jadwigi 27/39, 61-871 Poznań, Poland

**Keywords:** interleukin 11 (IL-11), bone metastasis, breast cancer, bone, osteolysis

## Abstract

Bone metastases represent the main problem related to the progression of breast cancer, as they are the main cause of death for these patients. Unfortunately, to date, bone metastases are incurable and represent the main challenge for the researcher. Chemokines and cytokines affect different stages of the metastatic process, and in bone metastases, interleukin (IL) -6, IL-8, IL-1β, and IL-11 participate in the interaction between cancer cells and bone cells. This review focuses on IL-11, a pleiotropic cytokine that, in addition to its well-known effects on several tissues, also mediates certain signals in cancer cells. In particular, as IL-11 works on bone remodeling, it plays a relevant role in the osteolytic vicious cycle of bone resorption and tumour growth, which characterizes bone metastasis. IL-11 appears as a candidate for anti-metastatic therapy. Even if different therapeutic approaches have considered IL-11 and the downstream-activated gp130 signaling pathways activated downstream of gp130, further studies are needed to decipher the contribution of the different cytokines and their mechanisms of action in breast cancer progression to define therapeutic strategies.

## 1. Introduction

Despite advances in cancer treatment, therapeutic options for bone metastases are still inadequate and, generally, palliative [1]. Furthermore, the prolongation of survival of cancer patients, due to the availability of effective therapies, is associated with the onset of bone metastases for neoplasms that rarely have bone as a secondary growth site [2,3].

Novel combination strategies that simultaneously target both primary tumour and bone metastasis are desirable to improve patient outcomes. This is particularly relevant for breast carcinoma, as bone metastases are responsible for 90% of deaths for mammary carcinoma [4].

Chemokine and cytokine signalling intervenes and regulates different steps of the metastatic process, starting from the detachment of tumour cells from the primary tumour mass to bone colonization, participating in the epithelial-to-mesenchymal transition (EMT), cell migration, seeding, and proliferation [5,6]. Considering the bone metastases, interleukin (IL)-6, IL-8, IL-1β, and IL-11 mediate the crosstalk between bone cells and tumour cells acting on bone homeostasis: they show a bone trophic function and are regulators of bone remodelling. In this context, the contribution of IL-11 appears peculiar, since it participates in the establishment of a vicious cycle of bone resorption and tumour growth, thus promoting bone colonization. Moreover, IL-11 is present in larger amount in cancer compared to IL-6 and, thus, it may play a relevant role in neoplastic disease [7].

In this review, we focus on IL-11, a member of the IL-6 cytokine family, which exerts pleiotropic effects in homeostasis and in disease. IL-11 mainly functions as an anti-inflammatory cytokine though it can act as a pro-inflammatory mediator, a feature shared with IL-6 [8]. The pleiotropic nature of this cytokine emerged early after its discovery: first identified as a stimulating factor for murine plasmacytoma cells [9], it was next described as secreted by bone marrow cell lines and able to inhibit differentiation of pre-adipocytes [10,11]. Since then, several biological roles have been attributed to IL-11. IL-11 is a powerful hematopoietic factor that, synergistically with other cytokines (e.g., IL-3, IL-4), induces megakaryocytopoiesis [12]. Further, IL-11 alone stimulates the recovery of platelets after radiation therapy, in mice [13], and its recombinant version has been approved by the FDA to treat radiation-induced thrombocytopenia in tumour patients (e.g., breast cancer) [14]. It is also involved in myelopoiesis, lymphopoiesis, and erythropoiesis [15]. At bone level, IL-11 has been shown to promote osteoblast differentiation in mice [16,17], while mutations in its sequence or in that of its receptor (IL-11Rα) have been associated with height growth deficit, [18], osteoarthritis [19], and craniosynostosis [20] in humans. Overall, the studies clearly indicate that IL-11 signalling is involved in growth regulation [15]. Moreover, IL-11 is implicated in reproduction: mutations in IL-11Rα, in female mice, associate with infertility [21] and, hence, it has been under investigation as a contraceptive [22]. IL-11, indeed, regulates the invasion of the extravillous trophoblast during placentation and seems to be also involved in the onset of preeclampsia [23]. Besides the physiological roles, the aberrant expression of IL-11 is associated with the evolution of several pathological conditions. For instance, its expression is increased in the case of viral-induced asthma [24], and in mycobacterium tuberculosis infection [25], in airways and lung where it is critical for the T-helper (Th2)-mediated inflammatory response [26] and, indeed, the inhibition of IL-11 signalling improves the inflammatory status [27]. IL-11 has a role in fibrotic degeneration [28] of different organs such as heart [29], liver [30], and lung [31,32] after chronic inflammation and failure. It is a downstream effector of transforming-growth factor (TGF)β and acts in an autocrine fashion [29].

The role of IL-11 in cancer has been the subject of extensive studies in view of the emerging evidence indicating IL-11 as a signalling mediator in cancer cells in defining worst outcomes. IL-11 is involved in different aspects of the tumorigenesis including proliferation, angiogenesis, survival under hypoxic condition, radio- and chemo-resistance, and apoptosis suppression [33,34,35,36]. IL-11 promotes tumorigenesis mainly by triggering the JAK-STAT3 pathway. Elevated IL-11 expression has been associated to various human cancers of both epithelial and hematopoietic origin. IL-11 is secreted not only from different types of cancer cells but also from cancer-associated cells, including cancer-associated fibroblasts and myeloid cells. In this way, the cytokine is able to participate in the bidirectional crosstalk tumour-microenvironment. IL-11 is produced by breast cancer cells and has been implicated in breast cancer-induced osteolysis. Moreover, the release of high levels of IL-11 by mammary tumour cells correlates with an elevated possibility to develop bone metastasis [37,38]. Due to its pro-tumorigenic activities, IL-11 signalling inhibition appears as a new strategy to be used in cancer therapy [39].

This review has the aim to take stock of the current knowledge about the role of IL-11 on bone metastasis development to highlight the therapeutic opportunities that the modulation of its activity may offer.

## 2. IL-11 Structure and Signal Transduction

IL-11 is a multifunctional cytokine member of the IL-6 cytokine family, which includes, other than IL-6, also the leukaemia inhibitor factor (LIF), oncostatin M (OSM), ciliary neurotrophic factor (CNTF), cardiotrophin-1 (CT-1), cardiotrophin-like cytokine (CLC), neuropoietin (NPN/CT-2), IL-27, and IL-31. Table 1 reports the main functions of the IL-6 cytokine family members [40,41].

IL-11 acts throughout the transmembrane glycoprotein β-receptor subunit (gp130), a co-receptor shared with all the family members [42]. Given this peculiarity, IL-11, while showing unique biological activities, partly exhibits functional overlaps with all the members. Within the family, however, only IL-11 and IL-6 utilize gp130 subunit in a homodimeric complex.

**Figure 1:**
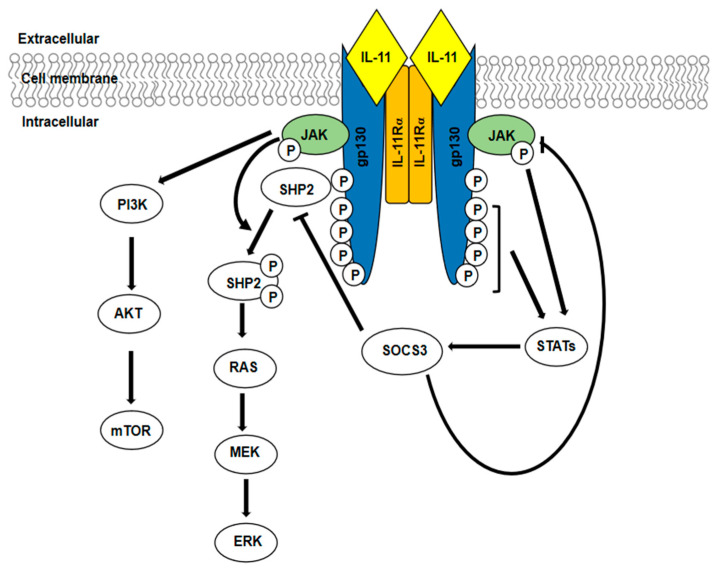
IL-11 signalling pathway. Upon the formation of the hexameric complex, the tyrosine phosphorylation of gp130 triggers the activation of three intracellular pathways: JAK/STAT, RAS/ERK, and PI3K/AKT/mTOR.

IL-11 signalling begins after the interaction of the cytokine with a specific membrane-bound α-receptor (IL-11Rα), which has only the function of binding the ligand. This event is followed by the engagement of gp130 (the signal-transducing β-receptor), the dimerization of gp130 subunits, and the formation of a hexameric complex: one gp130 homodimer plus two IL-11/IL11Rα couples. This hexameric complex activates the downstream signalling pathways, which, as for all the IL-6 family members, results in the activation of three major pathways: JAK/STAT pathway, Ras/Raf/MAPKs signalling cascade, and PI3K/AKT pathway (Figure 1). The formation of the hexameric complex activates the associated JAKs by trans-phosphorylation. Once activated, JAK kinases in turn phosphorylate various cytoplasmic tyrosine residues in gp130, generating docking sites for other signalling molecules and, thus, initiating distinct intracellular signalling cascades. The phosphorylation of STAT binding sites recruits STAT proteins, which are themselves phosphorylated. This event leads to STATs’ activation and their translocation to the nucleus where they act as transcription factors. Upon activation, gp130 offers also a binding site (Y759) for the Src homology-2 domain-containing phosphatase (SHP2) [43]. Activated JAK phosphorylates SHP2, which becomes able to recruit other signalling molecules leading to the activation of Ras/Raf/ERK pathway. IL-11 also activates the PI3K/AKT/mTOR pathway independently of Tyr phosphorylated gp130, event that requires JAK activation [39,44]. The duration of the receptor signal activation is regulated by suppressors of cytokine signalling (SOCS), negative regulator of gp130 signalling. SOCS3, which is transcriptionally regulated by STAT, binds the kinase domain of JAKs, while the direct binding of SOCS3 to gp130 (Y759) blocks cytokine receptor and mediates the receptor complex ubiquitination and degradation [45,46]. The induction of JAK-STAT3 pathway is of particular interest given its role in oncogenesis [47]. It has been reported that the activation of the JAK-STAT axis promotes cell migration and invasion. Indeed, EMT-related genes, such as matrix metalloproteinases (MMPs), are targets of STAT. MMPs play a relevant role in tumour growth and invasion by degrading extracellular matrix (ECM) and by allowing the release of growth factors and cytokines stored in ECM [48]. The JAK/STAT pathway sustains tumour growth also by promoting angiogenesis: vascular endothelial growth factor A (VEGFA) and hypoxia-inducible factor 1 α (HIF-1α) are target genes of STAT [39]. Moreover, PI3K-AKT-mTORC1 pathway, triggered by IL-11 signalling, contributes to tumour progression and metastasis. This pathway plays known anti-apoptotic and survival promoting roles in cancer [49], but it is also important as an inducer of invasion and proliferation of tumour cells acting downstream of IL-11 [50].

## 3. Regulation of IL-11 Expression in Physiological Conditions and in Cancer

### 3.1. Regulation of IL-11 Expression in Physiological Conditions

IL-11, physiologically expressed at low levels, is secreted by many cells with mesenchymal origin: chondrocytes, osteoblasts, leukocytes, fibroblasts, keratinocytes, synoviocytes, but also epithelial cells (the major source of IL-11). IL-11 is a non-glycosylated secretory protein of 178 amino acids, with a molecular mass of about 19 kDa; its gene is located on the long arm of the human chromosome, locus 19q13, the genomic sequence is of 7 kb and comprises 5 coding exons and 4 introns.

IL-11-promoter contains binding sites for activator protein-1 (AP-1), Runt-related transcription factor 2 (Runx2) as well as several Smad binding elements [51]. The transcription and subsequent expression of IL-11 appear to be mainly regulated by Extracellular-signal Regulated Kinases (ERKs) and transcription factors of the AP-1 family. IL-1β and TGF-β are known to induce IL-11 expression, which involves signalling of ERKs and p38 mitogen-activated protein (MAP) kinases via AP-1 [52,53]. TGFβ-triggered IL-11 secretion appears to be critically involved in the initiation of metastasis of colorectal cancer cells [54]. Of note, a long non-coding RNA activated by TGF-β (lncRNA-ATB) promotes organ colonization of disseminated hepatocellular carcinoma cells by binding the IL-11 mRNA and the autocrine induction of IL-11 expression [55].

### 3.2. Regulation of IL-11 Expression in Cancer

In vitro experiments reveal that TGFβ-dependent IL-11 induction is critical to provide bone metastases phenotype to breast cancer cells [56].

In bone disease associated to multiple myeloma, the production of IL-11 is stimulated by hepatocyte growth factor (HGF). In this context, IL-11 stimulates osteoclast (OC) recruitment and inhibits osteoblastic bone formation [57]. Moreover, the same authors reported that TGF-β1 and IL-1 potentiate the effect of HGF on IL-11 secretion, whereas an additive effect with TNFα is observed [57]. Even if these results derived from in vitro studies, they underline that a cytokine network works to make IL-11 available with important effects on aberrant bone resorption, which characterizes multiple myeloma.

Although IL-11 is virtually absent in body fluids of healthy individuals, its level increases in serum of patients in pathologic conditions, such as arthritis [58], acute pancreatitis, [59] pancreatic cancer [60], lipoedema [61], polycythaemia vera [62], lung disease in rheumatoid arthritis patients [63], and major cardiac events in chronic heart failure [64]. These results confirm a role for the cytokine in several pathological conditions, often in cancers and inflammatory diseases.

## 4. IL-11 and Bone

IL-11 is essential for physiological bone turnover and maintenance of bone structure. Once released by osteoblasts, IL-11 binds its receptor on both osteoblasts and osteoclasts, and in this complex microenvironment several signals regulate both IL-11 and IL-11R expression [65]. A role for IL-11 signalling in the bone development is suggested by in vivo and in vitro studies: IL-11 is able to promote bone formation in vitro [66], IL-Rα knockout mice show craniofacial abnormalities [17], the overexpression of IL-11, in transgenic mice, promotes bone formation [16], IL-11 stimulates osteoblastic differentiation in ST2 bone marrow stromal cells through STAT3-induced bone marrow morphogenetic protein 2 (BMP-2) [16]. IL-11 is also an osteoclastogenic cytokine [67]: a global deletion of IL-11R in mice determines low osteoclast number compared to control animals [17,68]. An essential role in physiological osteoclastogenesis has been observed also for the other members of the IL-6 family, which are able to stimulate osteoclast formation by promoting their production in osteoblast lineage cells [69]. IL-11 appears to stimulate osteoclastogenesis through a dual action: inhibition of osteoprotegerin (OPG) expression and induction of nuclear factor ligand-receptor κB (RANKL) activator production. The RANKL/OPG ratio may regulate the delicate balance between bone resorption and synthesis; OPG acts as a decoy receptor, binding to RANKL and blocking its interaction with RANK, thus inhibiting osteoclast development [70]. McCoy et al. reported that IL-11-induced osteoclast differentiation requires the presence of RANKL, which is released by osteoblasts [71]. Thus, this is a controversial issue: in fact, even if a functional role of IL-11 in the osteoclastogenic process has been well established, the exact mechanisms by which IL-11 promotes both the differentiation and function of osteoclasts warrant further analysis.

Based on these studies, it emerges that IL-11 governs bone remodelling and has a substantial impact on bone homeostasis. Thus, it becomes important to consider the functions of IL-11 in bone metastasis.

## 5. IL-11 and Bone Metastasis

Studies employing animal models have provided significant insights on the importance of IL-11 for cancer progression and it has been demonstrated that IL-11 drives metastasis in mouse models. As breast tumour cells are able to lead to bone disruption, when they grow in the bone metastatic site, several studies have considered the contribution of IL-11 in this condition. Of note, IL-11 is expressed by cells of the osteoblast lineage; therefore, cancer cells, other than producing IL-11, respond to this signal once they arrive in the bone marrow.

### 5.1. IL-11 and Breast Cancer Bone Metastasis: Data for Its Implications

Experimental evidence has shown a possible role of IL-11 in breast cancer bone metastasis. Breast cancer cells express IL-11R and secrete IL-11, which in turn stimulates osteoclasts [72,73]. In both human bone metastasis biopsies and experimental models, an increased osteoclast activity has been demonstrated close to the advancement margin of bone metastasis [74,75]. This led to the hypothesis that IL-11 may be associated with bone metastasis development in human breast cancer.

Sotiriou et al. demonstrated, for the first time, a significant enhancement of IL-11 expression in a cohort of 99 patients bearing primary invasive breast tumours and suggested the use of this cytokine as a predictive marker for the development of bone metastases [34]. In 180 breast cancer patients, IL-11 expression level was shown to be significantly increased in the serum of patients with bone metastases compared to patients without metastases, and this is associated with shorter overall survival [76]. These data parallel with augmented gp130 and STAT3 phosphorylation in tumour tissue, a pathway that leads to RANKL expression. The authors speculate that high levels of IL-11 promote bone degradation, which is responsible for the poor outcome. By acting on megakaryocytes, IL-11 stimulates the production of platelets and, indirectly, promotes metastases. Platelets can protect circulating cancer cells from attack by the immune system (immune evasion) and facilitate their arrest at the endothelium, supporting the development/establishment of secondary lesions [77]. Indeed, the number of megakaryocytes increases in the bone marrow of bone metastases-bearing animals [78] and, hence, it is conceivable that IL-11 supports this effect.

A positive correlation between the expression of IL-11Rα in tumour cells and bone metastasis incidence was reported in advanced breast cancer patients [79]. Lim et al. highlighted the functional role of hypoxia in the induction of IL-11 in breast cancer metastasis; IL-11 autocrine production plays an important role in cancer cell motility and invasiveness under hypoxic conditions [80]. Of note, since bone metastatic microenvironment is hypoxic, these results indicate that IL-11 is continuously produced. Hypoxia-induced IL-11 expression significantly alters EMT-related gene expression such as E-cadherin, N-cadherin, and vimentin, suggesting that the IL-11-STAT3 pathway may be involved in hypoxic tumour EMT [80,81].

As regards other bone-related cancers and their progression to metastasis, IL-11Rα is a cell surface marker of tumour progression and correlates with poor prognosis in patients with osteosarcoma [82]. The same authors show that IL-11Rα and its ligand, IL-11, are specifically upregulated in human metastatic osteosarcoma cell lines and that the engagement of this autocrine loop leads to tumour cell proliferation, invasion, and anchorage-independent growth in vitro.

**Figure 2:**
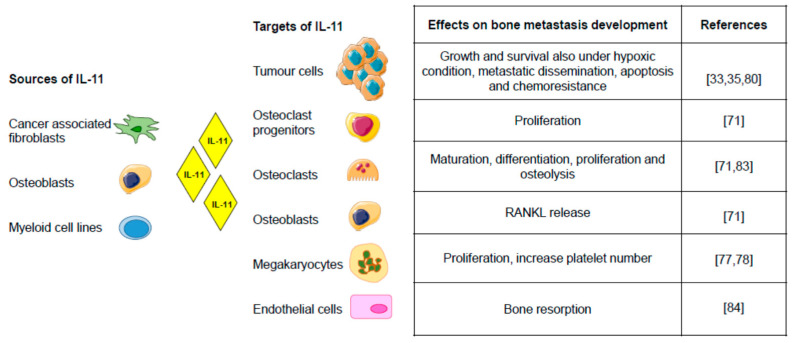
Sources, targets, and effects of IL-11 in bone metastasis [33,35,71,78,80,83,84]. IL-11 provides the cells a path to communicate with each other. It acts in paracrine as well as autocrine ways, contributing to the complexity of bone metastatic microenvironment (Figure created using Servier Medical Art available at https://smart.servier.com, accessed on 7 May 2021).

Figure 2 summarises the data of the IL-11 implication in bone metastasis.

### 5.2. IL-11 and Osteolysis in Breast Cancer Bone Metastasis

Kang Y et al., about twenty years ago, demonstrated that IL-11 is the most abundantly expressed osteolytic factor in breast cancer cells highly metastatic to bone, and that TGFβ further increases the already high level of IL-11 [85].

Breast cancer cell is known to produce numerous osteolytic factors, including parathyroid hormone-related protein (PTHrP), IL-1, IL-6, IL-8, IL-11, VEGF, connective tissue growth factor (CTGF), MMP1, HGF, etc. [86,87,88,89,90]. Some of these factors activate osteoclastogenesis by increasing RANKL expression in osteoblasts, while others activate osteoclastogenesis either synergistically to RANKL stimulation or in a RANKL-independent way. IL-11 released by breast cancer cells is able to stimulate osteoclast differentiation and increases osteoclast progenitor cells [71]. McCoy et al. characterized the role of IL-11 in osteoclast formation, function, and survival and indicated that primary function of IL-11 is related to the promotion of osteoclastogenesis by increasing the pool of osteoblast progenitor cells and by downregulating granulocyte-macrophage colony-stimulating factor (GM-CSF) expression. The authors specify that IL-11 produced by breast cancer cells induces osteoclast formation and bone resorption by two mechanisms, the first related to the production of RANKL by stromal cells/osteoblasts, and the second related to the rise of osteoclast progenitor through IL-11 derived from tumour cells. This partially explains the role of IL-11 in the promotion of osteolysis [71].

Liang M et al. demonstrated, by in vitro and in vivo experiments, that IL-11 plays an essential role in the vicious osteolytic cycle by activating osteoclastogenesis regardless of RANKL, via c-Myc activated by JAK1/STAT3 pathway in bone metastasis. Indeed, in vivo, the blockade of STAT3 phosphorylation results in the inhibition of osteolysis, and tumour growth of metastatic breast cancer [83]. Several studies have demonstrated that breast tumour cells can also target osteoblasts to stimulate the production of IL-11 [91,92], further increasing IL-11 concentrations in the bone microenvironment. Therefore, the production of IL-11 by cancer cells within bone can be direct or indirect, and, in turn, IL-11 inhibits bone formation by suppressing osteoblast activity [93]. Lysophosphatidic acid (LPA), a bioactive phospholipid derived from platelets and also present in tumour microenvironment, is known to play a critical role in in breast cancer osteolytic bone metastasis [94,95]. In an in vitro experiment, it has been reported that LPA enhances breast cancer cell-mediated osteoclastogenesis by inducing the secretion of osteolytic cytokines, such as IL-8 and IL-11. In particular, LPA induces IL-11 expression in MDA-MB231 breast cancer cells and this process seems to be related to the involvement of the PKCδ signalling pathway [96].

Prostaglandins (PGs) are abundant in bone, where they are released by cells of the osteoblast lineage and regulate bone metabolism. PGE2, a potent stimulator of osteolysis, represents a mediator of IL-11-induced bone resorption. Therefore, cyclooxygenase (COX)-2 inhibitors could be interesting drugs that potentially interfere with IL-11-mediated osteolytic metastasis. Several authors have, indeed, proposed COX-2 inhibitors (NS398, indomethacin, and dexamethasone) as drugs useful for suppressing IL-11-mediated osteolytic bone metastases of tumour cells [97]. Singh et al., focusing on the relationship between COX-2 and IL-11 in vitro both in poorly metastatic (MCF-7) and highly metastatic (MDA-MB231) breast cancer cell lines, demonstrated that COX-2 overexpression induces PGE2-mediated IL-11 expression, in both cell types. In an in vivo mouse model of breast cancer bone metastasis obtained with mice injected with COX-2-transfected MDA-435S cells, they isolated one-seeking clone from long-bone metastases that produces higher levels of PGE2 and correspondingly higher levels of IL-11 compared to COX-2-transfected parental MDA-435S cells. The authors demonstrated the important role of COX-2-mediated production of IL-11 in breast cancer cells and hypothesized the importance of this process in the development of osteolytic bone metastases in breast cancer patients. Therefore, they propose that COX-2 targeting may be useful in inhibiting the osteolytic process [98].

It has been reported that endothelial cells in bone play important roles in the promotion of bone resorption by secreting IL-11 in physiological and pathological conditions. In particular, bone-derived endothelial cells (BDECs) are specifically involved in bone osteolytic bone metastasis and also express the IL-11Rα and gp130, being in turn affected by IL-11 [84].

**Figure 3:**
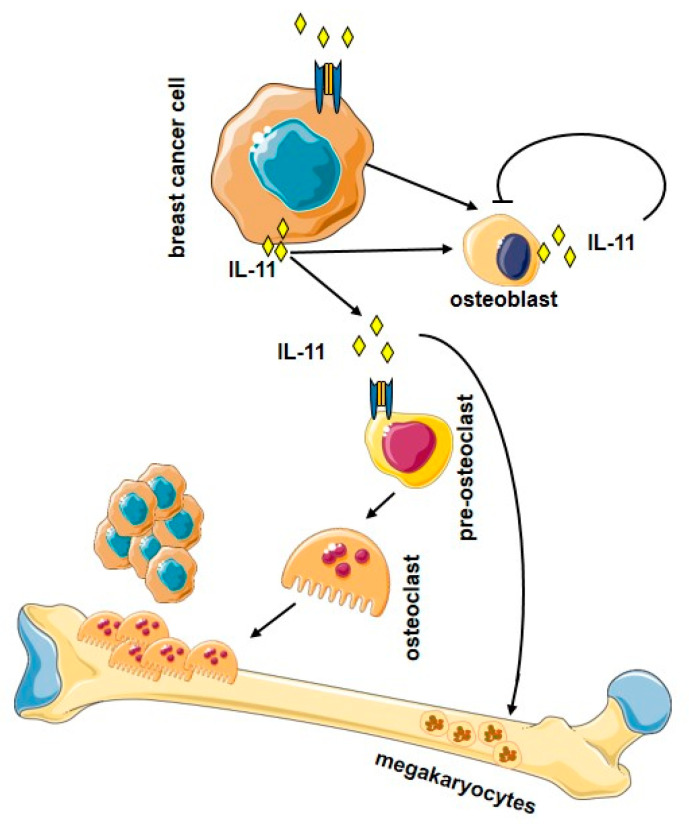
Overview of the osteolytic effects of IL-11 in bone metastasis. IL-11 released from breast cancer cells targets osteoblasts and osteoclasts. (Figure created using Servier Medical Art available at https://smart.servier.com, accessed on 7 May 2021).

Figure 3 illustrates the osteolytic effects of IL-11 in bone metastasis.

## 6. Therapeutic Strategies Targeting IL-11

The involvement of IL-11 in different stages of cancer development and progression has spurred many studies aimed at counteracting IL-11 itself or its signalling with different molecular approaches. Drug strategies aimed at targeting IL-11 fall mainly into two categories: monoclonal antibodies directed against either the cytokine or its receptor and small molecules that interfere with the receptor-signalling complex, including the gp130 or the downstream pathway of JAK-STAT [15,40]. Targeting human IL-11, or its signalling, in different types of cancer has been reported in few preclinical models [35,99,100,101,102]. In vitro experiments utilizing IL-11-neutralizing antibody were reported in breast cancer cells [71,80] with promising results. Among the small molecules, Bazedoxifene (a selective oestrogen receptor modulator) represents a potential molecule to be used as an inhibitor of IL-11 signalling. It binds gp130 and inhibits the downstream signal transduction, blocking STAT3 activation in human cancer cell lines. This effect results in the suppression of gp130-dependent tumour growth of the gastrointestinal epithelium [103]. IL-11 Mutein is a recombinant protein bearing a number of mutations generated to disrupt IL-11 signalling [27,104]. IL-11 Mutein, which binds the IL-11R with higher affinity than IL-11 (20 times more efficiently), showed positive results in the treatment of gastrointestinal cancers, in xenograft model [35]. Given that IL-11 Mutein does not show adverse effects on platelet numbers and it is well tolerated, it was suggested its potential use in clinical therapy also in other cancer types. In xenograft models of human endometrial cancer, it was reported the use of neutralizing anti-human IL-11R: the treatment transiently decreases tumour growth in mice inoculated with Ishikawa cells, while it reduces the dimension of tumour in mice injected with HEC1A cells [102]. Recently it has been reported a role of Asperolide A, a dipertenoid derived from marine algae, in the prevention of breast cancer bone metastasis. This agent, acting on PI3K/AKT/mTOR signalling cascade, efficiently inhibits osteoclastogenesis and prevents breast cancer-induced bone osteolysis [105]. Of note, Asperolide A intervenes on a pathway triggered by IL-11. Conversely, Oprelvekin, a recombinant human IL-11, is now routinely used to treat thrombocytopenia in breast cancer patients, which underwent radiation therapy, as an alternative to platelets transfusion. Due to its haematopoietic and megakaryocytopoietic activities, Oprelvekin reduces severe thrombocytopenia and accelerates platelet recovery [14,106].

Also, naturally occurring compounds may affect the IL-11 signalling. In traditional Indian and South East Asian medicine, Curcumin, derived from *Curcuma longa*, has been used to treat a variety of diseases given its known anti-inflammatory and anticancer properties. Indeed, Curcumin has shown the ability to modulate a lot of signalling pathways, among which STAT3 activation, the PI3K/AKT/mTOR, and HIF-1 signalling pathways [107]. Moreover, in an in vivo experiment, a preparation containing the essential turmeric oils in addition to the standard Curcumin demonstrated a higher bioavailability, a prerequisite to be adsorbed following the ingestion, resulting in upregulation of the expression of IL-10 and IL-11 [108]. Taken together, this preclinical and clinical evidence allow considering Curcumin and its derivatives as a tool to potentiate the chemotherapeutic agents against cancer.

### miRNA and Inhibition of IL-11 Signalling

Some miRNAs, a class of key posttranscriptional regulators [109], have been described to inhibit IL-11 signalling in different diseases as well as in breast cancer.

Pollari and colleagues elucidated the role of miRNAs in the bone metastatic process of breast cancer and specifically analysed the miRNAs that regulate TGFβ-induced IL-11 expression [110]. These authors identified miR-204, -211, and -379 as the strongest modulators of IL-11 production, these miRNAs directly downregulate a key pathogenetic process in breast cancer metastasis, i.e., the TGFβ-induced expression of IL-11.

Other authors reported that miR-124 inhibits breast cancer bone metastasis through the repression of IL-11 [111]. In this study, the authors demonstrated that miR-124 negatively regulates IL-11 expression, both in vitro and in vivo. A negative correlation has been demonstrated between miR-124 level and IL-11 expression, both in cell lines and in human metastatic bone tissues. Patients with lower miR-124 expression or higher IL-11 expression in metastatic bone tissues have a rapid progression of the disease and therefore a shorter overall survival. The authors propose miR-124 and IL-11 as new therapeutic targets for breast cancer patients at an early stage and prognostic markers in advanced stage patients with bone metastasis.

Bockhorn et al. have identified IL-11 as a relevant downstream target of twinfilin (TWF1), an actin-monomer-binding protein. TWF1 regulates the expression of IL-11 at both mRNA and protein levels. The authors demonstrated that miR-30c regulates breast cancer chemoresistance and EMT by direct targeting of the cytoskeleton gene TWF1 and thus by indirect targeting of the cytokine IL-11 [112].

Samaeekia et al. reported that miR-206 suppresses breast tumour stemness and metastasis by inhibiting both self-renewal and invasion. In triple-negative breast cancer cells, the authors identified the pathway mediated by miR-206: it targets TWF1, megakaryoblastic leukaemia (translocation) 1 (MKL1), and serum response factor (SRF), and subsequently leads to lower levels of IL-11 mRNA and protein expression [113].

## 7. Conclusions

IL-11 appears as a multifunctional cytokine with known roles on cancer (clearly pro-tumorigenic and pro-metastatic). Considering that, to date, the treatment for bone metastases is largely palliative, the fact that the cytokine performs an osteolytic action in these conditions places it at the center of investigations with the aim of finding solutions to improve the conditions of these patients. However, despite the promising results in animal models, IL-11-based therapies still have to overcome numerous challenges. The main challenge, in fact, is the collateral interference induced by the blockade of IL-11 or IL-11 signalling molecules, with the physiological mechanisms. Of note, in vivo, a plethora of signals, emanating from cancer/microenvironmental cells and influencing each other, works in the complex bone milieu. Many of these signals find their receptor on bone-resident cells and so they act to remodel bone. In this regard, some results collected with in vitro experiments to explain the mechanism of action of a particular cytokine may not be able to mimic a real situation that occurs in vivo. Deciphering the roles of IL-11 and other cytokines as well as of the intracellular signals involved in the colonization of bone metastases will lead to the identification of targets for coordinated therapies.

A deeper understanding of the role played by IL-11 in the various stages of tumour development and progression, in parallel with an improvement in the knowledge of the IL-11 signalling, will fully reveal its potential uses. Interesting and promising are some miRNAs, uncovered as key cellular mediators of the metastatic process in breast cancer in vitro and with potential clinical relevance to prevent or eventually treat breast cancer bone metastases. Therapeutic options for breast cancer patients at risk of progressing to bone metastasis are necessary, but IL-11-based therapy requires more extensive analyses to confirm and extend its use.

Finally, conflicting results are reported on the correlation between IL-11 expression and the receptor endowment of breast cancer cells [36,114]. This represents an important issue and further studies should be conducted to highlight the possible impact that IL-11 could have on the molecular classification of breast cancer as a prognostic marker.

## Figures and Tables

**Table 1:** Main biological functions of IL-6 family members.

Cytokines	Biological Functions
Interleukin 6	Acute phase response Haematopoiesis Angiogenesis Immune reactions Bone, glucose and lipid metabolism Oncogenesis
Interleukin 11	Haematopoiesis Megakaryopoiesis Growth factor activity Bone metabolism Oncogenesis
Interleukin 27	T cells differentiation Glucose metabolism Immune regulation Antitumor activity
Interleukin 31	Skin immunity
Oncostatin M (OSM)	Haematopoiesis Bone and lipid metabolism Regulation of cytokine production
Leukemia inhibitory factor (LIF)	Haematopoiesis Bone metabolism Neuronal cell differentiation
Ciliary neurotrophic factor (CNTF)	Haematopoiesis Bone metabolism Growth and differentiation activities Neurotrophic factor
Cardiotrophin-like cytokine (CLC)	Development of nervous system B-cell stimulation Haematopoiesis Immune cell function
Cardiotrophin 1 (CT-1)	Cell proliferation Muscle and nervous system development Cardioprotective effects Energy metabolism Hepatoprotective effects
